# Evaluation of Wedged Arterial Injection as a New Technique for Delivery of Experimental Therapeutic Sustances into the Porcine Pancreas

**DOI:** 10.1155/2011/976910

**Published:** 2011-10-04

**Authors:** Rafael Latorre, Wendy Hernández, Fei Sun, Francisco Sánchez-Margallo, Francisco Gil, Octavio López-Albors, Jose M. Vázquez, Jesús Usón

**Affiliations:** ^1^Anatomy and Embryology, Veterinary Faculty, University of Murcia, Campus de Espinardo, 30100 Murcia, Spain; ^2^Jesús Usón Minimally Invasive Surgery Centre, 10071 Cáceres, Spain

## Abstract

*Objectives*. To prospectively evaluate the technical feasibility and efficacy of wedged arterial injection (WAI) as a potential route for experimental selective therapy to the pancreas of healthy pigs. *Materials and Methods*. Selective angiographies were completed in ten pigs under general anaesthesia. By superselective angiography, the catheter was inserted and wedged into the major pancreatic artery, blocking the blood flow. In order to evaluate the efficacy of the WAI method, a DNA-specific fluorescent dye (Hoechst 33258) was used. *Results*. Histological study revealed a uniform distribution of the fluorescent dye within the nuclei of the endocrine and exocrine pancreatic cells. Pancreatic and liver enzymes as well as histopathology of the pancreas were normal. *Conclusion*. WAI is a highly effective minimally invasive methodology to target the porcine pancreas. The findings suggest that WAI may contribute to developing preclinical assays of pancreas gene or cell-transfer therapies in swine model.

## 1. Introduction

Animal models are essential tools for investigating the aetiology and pathogenesis of diabetes as well as for developing effective treatment methods, such as islet transplantation or genetic engineering [1, 2]. Although a range of well-characterized and widely used models of type 1 diabetes in rodents are currently available, they do not reliably predict therapeutic outcomes in larger mammals [2–4]. Large animal models are a valuable complement to rodents for both physiological and practical reasons. Anatomically, the porcine and human pancreases are quite similar. In both species, it is a retroperitoneal organ, and the pancreatic head wraps the portal vein [5]. In addition, the firmness of the pig pancreatic parenchyma is similar to the gland in humans [6]. Physiologically, pig islets offer a very competitive product for *β*-cell replacement because of their morphological characteristics, ability to respond to glucose challenge, and cell composition and also because of the fact that the pig and human insulin differ in only one amino acid [5, 7].

Among the new emerging technologies for diabetes therapy, gene and cellular injections are currently attractive and of great potential. However, there are persistent and severe obstacles to their applications mainly because of the topography and structure of the pancreas [3]. So far, there are no effective ways of approaching the pancreas without causing significant unwanted effects, such as infection of other organs [8] or toxicity and damage of the pancreas parenchyma [3, 9]. Arterial or venous injection via the pancreatic circulation has been reported in dogs [10], but surgical clamping of the pancreatic circulation was necessary. Alternatively, gene injection through the pancreatic duct has been tested in rats and mice [3], but the high infusion pressure required was related to complications like pancreatitis. 

Successful islet gene and cellular therapies will depend on the appropriate choice of the route of administration [2, 10]. To make these therapies viable in the clinical setting, preclinical investigations should be aimed at identifying safe ways to deliver the cells or vectors into recipients and at defining effective methods for preventing postadministration loss of transferred elements. To our knowledge, no previous experimental studies involving large mammals have been reported on this subject. Therefore, our objective was to prospectively evaluate the technical feasibility and efficacy of wedged arterial injection (WAI) into the pancreas of healthy pigs as a potential route for future selective diabetes therapy.

## 2. Materials and Methods

### 2.1. Animals

All animals received humane care in compliance with the European Communities Council Directive (86/609/EEC). Protocols were approved by the ethics committee for animal research of the Jesús Usón Minimally Invasive Surgery Centre. Five fresh cadavers of large white pigs weighing 48.2 ± 3.6 kg were used for anatomic vascular study. The reason for euthanasia was unrelated to pancreatic disorders. Additionally, ten healthy Large White pigs (five female and five male) of 54.8 ± 5.3 kg were randomly distributed into group 1 (*n* = 5, sacrificed 1 hour after procedure) or group 2 (*n* = 5, sacrificed 7 days after procedure). In all these animals, selective angiographies followed by WAI of Hoechst 33258 (fluorescent dye) were performed. CT angiography was carried out before and after procedure in each animal.

### 2.2. Vascular Anatomy

The arterial and venous systems of cadavers were injected with red- and blue- colored epoxy resin, respectively (Araldite CY 223-HY 2967). Four months after injection, corrosion casts were cleaned with running tap water and careful dissection. The pancreatic vascular supply was identified, and the diameter of the main pancreatic artery, which supplies the left lobe, was measured at its origin and 4, 6, and 8 mm further by using a stereoscope (Stemi DV4, Carl Zeiss).

### 2.3. Angiography

All pigs were fasted for 24 hours before medication with 0.1 mg of diazepam per kilogram of body weight, 10 mg/kg ketamine, and 0.01 mg/kg atropine intramuscularly. General anesthesia was induced with 2 mg/kg propofol administrated intravenously. After endotracheal intubation, pigs were connected to an anesthesia system (Ohmeda Excel 210 SE; Boc Group, Madison, Wis, USA) and mechanical ventilator (Ohmeda 7800; Boc Group). Anesthesia was maintained with 2%–2.5% halothane. Animals were positioned in supine recumbency and given 0.15 mL of amoxicilin trihydrate plus potassium clavulanate (Synulox; Pfizer, New York, NY, USA) per kilogram of body weight intramuscularly.

A CT angiogram (Philips Brillance CT-6) with vascular 3D reconstruction was obtained from each animal so as to monitor the individual vascular anatomy before angiographic procedure. 

The groin area and lower abdomen were then sterilely shaved and draped. Systemic heparinization was obtained by intravenous administration of 150 IU/(Heparina ROVI 0.5%, ROVI SA, Madrid, Spain) per kilogram of body weight. Percutaneous femoral arterial access was gained with a 5-French introducer sheath (Check-flo; William Cook Europe, Bjaeverskov, Denmark). Under fluoroscopic guidance (BV-300; Philips, Best, the Nertherlands), a 0.035-inch hydrophilic guide wire (Radifocus guidewire, Terumo, Tokyo, Japan) and a 5-French cobra catheter (Radiofocus Cobra Small; Terumo, Tokyo, Japan) were placed into the aorta. After abdominal angiography was performed, selective catheterization of the celiac artery was achieved in the posterior-anterior view with 15 mL, of Urografin (Urografin, 76%, Schering Inc., Germany). Celiac arteriograms clearly depicted the vascular anatomy of the hepatic and splenic arteries and their branches. By road-map guidance, the cobra catheter was selectively positioned and wedged into the major pancreatic artery. A superselective angiography of this artery was conducted by manual injection of 3–5 mL contrast medium to ensure that the tip of the catheter was at the desired site. In order to evaluate the efficacy of the WAI method, a DNA-specific fluorescent dye (Hoechst 33258: Bisbenzimide H33258 Fluorochrome Trihydrochloride. Calbiochem, Merck KGaA, Germany) was used. WAI of 2 mL 0.5% Hoechst 33258 diluted in phosphate buffered saline (PBS) was slowly injected (2 minutes) under fluoroscopy guidance. During WAI of Hoechst and during the following 15 min the infusion catheter was wedged so as to block the blood flow to the pancreas. Then, the catheter and the introducer sheath were removed from the femoral artery and haemostasis was achieved.

### 2.4. Postprocedure Evaluation

Blood samples were taken just before (groups 1 and 2) and 7 days after procedure (group 2) and used to determine the serum concentrations of amylase, lipase, C-reactive protein (CRP), alanine aminotransferase (ALT), and aspartate aminotransferase (AST). These enzymes were used as indicators of potential pancreatic and liver damage. 

Hoechst staining was evaluated in animals from group 1. Euthanasia was carried out by intravenous injection of potassium chloride solution. Necropsy and gross pathologic examination were immediately performed. The pancreases were removed, and 25 histological samples were obtained from the three pancreatic portions (body, and right and left lobes). Samples were coated in embedding compound (Tissue Freezing medium, Leica Instruments, GmbH Nussloch, Germany), frozen by 20 seconds immersion in liquid nitrogen, and stored at −70°C until analysis. Sections (10 *μ*m thick) of each sample were cut at −25°C using a cryostat (Reicher Jung Criocut). Fluorescence microscopy (excitation 365 nm, emission 450 nm) was used to evaluate nuclear DNA staining with Hoechst-33258. 

Group 2 pigs were allowed to recover from general anaesthesia and observed for signs of decreased activity, irritability, vomiting, or anorexia twice daily. On day 7, animals were euthanized with intravenous injection of pentobarbital overdose. Pancreases were examined in situ and removed, and 30 samples per animal of approximately 1 cm^3^ from the body and the two lobes were fixed in 4% paraformaldehyde. The samples were then paraffin-embedded and stained for haematoxylin-eosin so as to evaluate the histology of the pancreas.

### 2.5. Statistical Analyses

All data were included in a worksheet and analysed with the statistic package SPSS 17.0 (SPSS Inc, Chicago, Ill, USA). Variables tested for normality (Kolmogorov-Smirnov test) and descriptive statistics were obtained. The general linear model (Anova) of repeated measures was used to test the levels of enzymes in the blood samples, with two within subject factors (baseline and day 7).

## 3. Results

### 3.1. Vascular Anatomy (Figures 1(a)–1(d))

**Figure 1:**
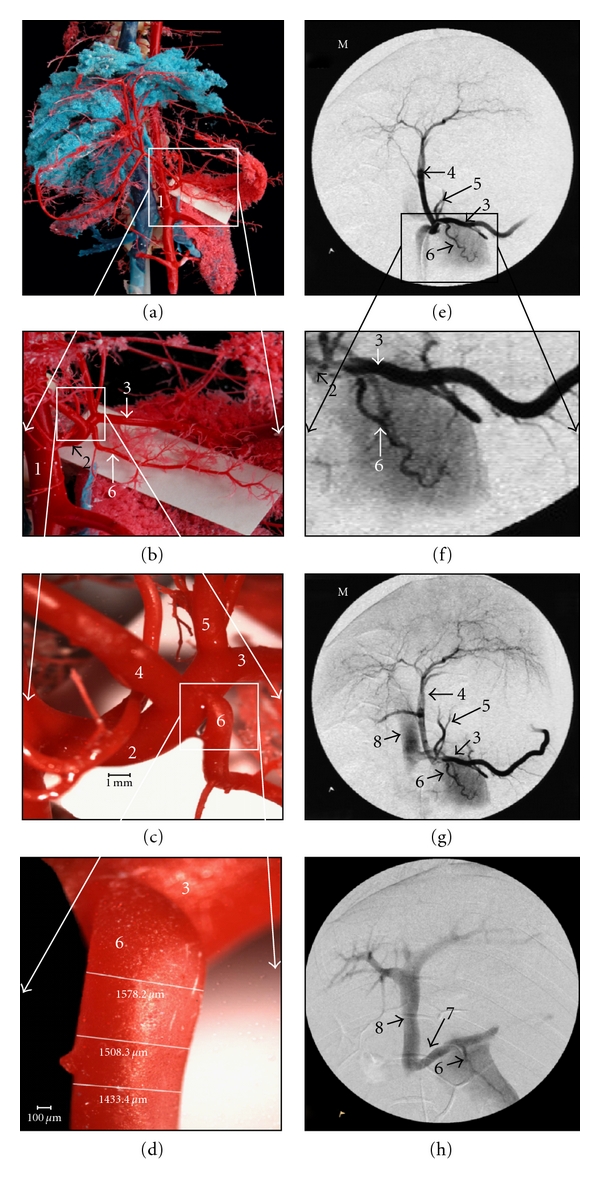
(a) Corrosion cast of the pig abdominal cavity. (b) Magnification from the square in previous image. (c) Magnification from the square in previous image, detail of the celiac artery branches. (d) Magnification from the square in previous image, detail of the major pancreatic artery. (e) Selective celiac angiography. (f) Magnification from the square in previous image, detail showing the major pancreatic artery from the splenic artery. (g) Selective celiac angiography, with contrast in portal vein. (h) Superselective angiography of the major pancreatic artery. (1) Abdominal aorta; (2) Celiac artery; (3) Splenic artery; (4) Hepatic artery; (5) Left gastric artery; (6) Major pancreatic artery; (7) Pancreatic vein; (8) Portal vein.

Corrosion casts demonstrated that the right lobe and body of the pancreas are supplied by a vascular arcade resulting from anastomosis of the cranial and caudal pancreaticoduodenal arteries. The left pancreatic lobe, however, is supplied by a single, terminal, and constant major pancreatic artery (Figure 1(a)), which arose from the first portion of the spleen artery in 4 out of 5 pigs (80%) and from the hepatic artery in one pig (20%). The average diameters of this artery at its origin and 4, 6, and 8 mm further were 2.07 ± 0.025 mm, 1.574 ± 0.09 mm 1.581 ± 0.03 mm, and 1.395 ± 0.04 mm, respectively.

### 3.2. Angiography

3D reconstruction of CT angiograms obtained before angiography was useful to identify the origin and potential anatomical variation of the major pancreatic artery supplying the left pancreatic lobe.

Selective catheterization and angiography of the celiac artery were successfully achieved in pigs of both groups by using a 5-French cobra angiographic catheter. The angiograms confirmed the sole participation of the major pancreatic artery in the blood supply of the left pancreatic lobe (Figures 1(e)-1(f)). This artery arose from the splenic artery in 8 pigs (80%) and from the hepatic artery in 2 pigs (20%). At its origin, the artery's diameter was slightly larger than that of the catheter tip (1.67 mm). This allowed the angiographic catheter to be inserted and wedged within the main pancreatic artery a few millimetres distal of its origin. Consequently, blood flow towards the left lobe of the pancreas was completely blocked after the catheter was wedged within the artery. Superselective angiography was performed before the injection of Hoeschst. The contrast medium stains the parenchyma of the left lobe of the pancreas, being ultimately drained by the spleen and the portal veins (Figure 1(g)).

Significantly, no procedure-related complications, such as thrombosis, spasm of the target artery, or intimal dissection were noticed during WAI.

### 3.3. Postprocedure Observation

**Figure 2:**
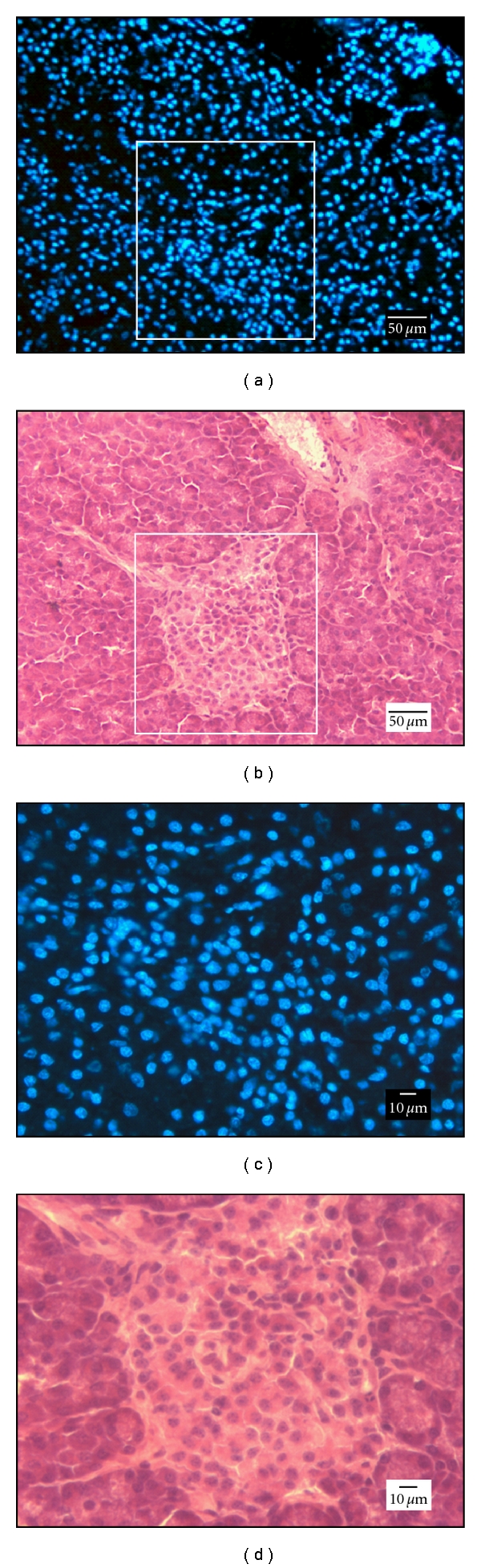
Analysis of tissue after wedged arterial injection of Hoescht. Fluorescence image of cryosection from left lobe of the pancreas showing homogenous distribution of endocrine and exocrine cells with nuclei stained and serial section stained with hematoxyline-eosine.

Localization of the Hoechst dye within the nuclei of endocrine and exocrine pancreatic cells of the left pancreatic lobe confirmed that WAI was successful (Figure 2). However, no Hoechst positive cells were observed in histological samples from the body and right pancreatic lobe. 

All animals tolerated the procedure without clinical symptoms of acute pancreatitis or distress. In group 2, during the 7-day observation period, behaviour and dietary intake were normal in all pigs. Besides, blood levels of pancreatic and liver enzymes did not reveal significant differences between the baseline and values recorded 7 days after procedure (Table 1). At necropsy, the pancreatic gland showed normal without fluid collection, abscess, or hemorrhage. Histological study did not revealed any evidence of pancreatitis or abnormalities of the pancreatic duct system.

## 4. Discussion

Different ways of delivering therapeutic agents into the pancreas have been described in rodent models [3, 8, 9] and large mammals, such as the dog [10] and the monkey [11]. Success of “in vivo” experimental pancreatic therapy largely depends on the use of an appropriate route of administration [2, 10]. It should be safe and super selective to the pancreatic parenchyma, thus impeding the loss of the transferred elements. Assay of pancreatic islet transplantation through the celiac trunk carried out in nonhuman primates has been described as a less effective method [12]. The absence of islets in the pancreases of the transplanted primates could be related to the nonuse of a superselective approach through a pancreatic artery.

 In this paper, a superselective approach to the left pancreatic lobe of pigs was tested by wedged arterial injection (WAI) method. WAI was carried out through a terminal and single vessel, the major pancreatic artery, which in pigs supplies the left pancreatic lobe. This artery resembles the great pancreatic artery of humans although the major pancreatic artery of pigs does not anastomose with other pancreatic arteries. While the major pancreatic artery is not mentioned in the Nomina Anatomica Veterinaria [13], it has been commonly described in other published reports on the vascular anatomy of the swine pancreas [5, 6, 14–16]. Meticulous dissections allowed the description of an anatomical variation concerning its origin, which was referred to being from the spleen artery (85.5%) or the hepatic artery (14.5%) [14]. These results are very similar to the respective 80% and 20% found in the present study. However, the work of Traverso and McFarlane [6], whose anatomical variation refers to the origin of the major pancreatic artery from the celiac trunk, was not found in any of the animals used in this work. A possible reason for this discrepancy might be the sample number or the use of different pig breeds. 

The diameter of the major pancreatic artery was independent of its origin from the spleen or hepatic artery (2.07 ± 0.25 mm). This size was wide enough for placement of the 5 French angiography catheter, thus supporting the technical feasibility of the WAI method as a minimally invasive vascular approach to the pancreas. In contrast, using classical surgical techniques the access to the pancreatic arteries is rather difficult, as the pancreatic vessels tend to run within the pancreatic tissue, and the pancreas itself has a complicated surgical approach. That was the case of a previous study in dogs [10], where laparotomy and manual clamping of the pancreatic vessels were necessary for an effective vascular delivery of genes within pancreatic parenchyma.

There is significant motivation to develop effective methods for preventing postinjection loss of infused elements during pancreatic therapy [2]. After intravascular infusion, it is imperative to ensure the elements to stay in contact with the pancreatic cells for as long as possible. Using the WAI method, as performed in this study, the elements (drugs, virus, etc.) can be slowly and directly injected into the pancreas. The average diameters of the major pancreatic artery 4, 6, or 8 mm distal to its origin were smaller than the 5 French catheter tip (1.67 mm), and this made possible a blockage of the blood flow by wedging the artery lumen with the catheter tip. This allowed a slow delivery of the Hoechst dye all along the left pancreatic lobe, avoiding, at the same time, being washed out by the blood flow. Giving of future potential therapeutic applications, this assures an extended contact time between the infused therapeutic agents and the pancreatic tissue. 

Bisbenzimide H 33258 Fluorochrome (Hoechst) has been used in this study as a marker to demonstrate the efficacy of WAI to transfer drugs into the pancreatic tissue. Hoechst is a membrane-permeable, adenine-thymine-specific fluorescent dye useful for in vivo staining of DNA. Intravenous in vivo injection of Hoechst has been used extensively for tumour cell labelling [17–20] as well as to determine the vascular perfusion of pancreatic tumours [21] and acute hypoxia [22]. In this work, positively stained pancreatic cells were only found in the left pancreatic lobe, that is, the potential distribution area for drug infusion when a superselective WAI through the major pancreatic artery is performed. Conversely, no positive Hoechst cells were found in the body or right pancreatic lobe, and this might be relevant, because the left pancreatic lobe may accomplish up to the 70% of the total organ weight [5]. In a similar way, a delivery of anticancer agents through a catheter superselective to the great pancreatic artery has been reported to improve the therapeutic effects of chemotherapy for the human pancreatic carcinoma [23].

A relevant finding of this study was that no adverse effects related to the procedure were found in the pancreatic function and structure. The level of pancreatic and liver enzymes did not increase significantly between the baseline and 7 days after procedure. Consequently, no functional alterations of the pancreas or liver are likely to be expected after WAI. Structural disorders of the pancreatic parenchyma were not observed either. All pancreases displayed normal architecture, independently of the sampling site. Accordingly, WAI through the major pancreatic artery was not only a minimally invasive technique but also a safe approach to the pancreatic parenchyma in the porcine model. 

Nevertheless, further experiments aimed at gene transferring or cell transplantation are required. In this sense, gaining a precise knowledge of the biodistribution of vectors injected by WAI is of priority.

### 4.1. Practical Applications

The reported WAI method for a minimally invasive approach to the porcine pancreas by using interventional radiology techniques was effective, safe, and reproducible. The described WAI method might contribute to further developing diabetes therapies in swine models.

### 4.2. Summary Statement

Wedged arterial injection (WAI) is proposed as a valuable minimally invasive approach to the porcine pancreas. WAI is a safe technique that may contribute to assay experimental diabetes therapies in swine model.

## Figures and Tables

**Table 1:** Enzyme levels (U/L) in blood samples before and after procedure. Values are average ± standard deviation. CRP, C-protein reactive; ALT, alanine aminotransferase; AST, aspartate aminotransferase.

	Baseline	7 days	*P*
Amilase	1339.34 ± 141.7	1652.24 ± 38.46	0.13
Lipase	4.64 ± 0.49	6.18 ± 2.24	0.202
CRP	32.86 ± 16.09	58.6 ± 24.06	0.268
ALT	33.1 ± 2.97	35.22 ± 1.36	0.618
AST	29.52 ± 1.4	40.1 ± 5.1	0.16
